# Late-Life Cardiac Interventions and the Treatment Imperative

**DOI:** 10.1371/journal.pmed.0050007

**Published:** 2008-03-04

**Authors:** Janet K Shim, Ann J Russ, Sharon R Kaufman

## Abstract

The treatment imperative, say the authors, refers to the almost inexorable momentum towards intervention that is experienced by physicians, patients, and family members alike.

In this Essay, we offer reflections based on our previously published ethnographic study of physician and patient responses to life-extending cardiac procedures that are increasingly performed on ever older individuals in the United States [1]. In that study, we examined how providers and patients considered advanced age in their decision-making about various treatments (angioplasty, stents, bypass surgery, and implantable cardioverter defibrillators), using in-depth interviews with physicians from internal medicine and cardiac subspecialties, cardiac patients aged 72–86, and their family members. The project received institutional review board approval from the University of California, San Francisco, and all interviewees gave informed consent prior to participation.

Despite evidence indicating that elderly patients tend to receive more conservative cardiac treatment than may be medically indicated [2,3], clinicians, patients, and family members in our study all perceived a nearly inescapable obligation to pursue cardiac interventions. We summarize here our findings on the socio-medical sources of this treatment imperative, and its impacts on physicians' and patients' encounters with life-prolonging cardiac procedures. Based on our study findings, we then offer several recommendations regarding patient–provider communication that seek to improve the experiences of elderly patients, and perhaps those of physicians as well.

## The Treatment Imperative as a Clinical–Ethical Obligation

Underlying the treatment imperative are at least three changes in the socio-clinical landscape of cardiac intervention in the US. First, the social meaning of cardiac treatments has shifted, from aggressive, high-risk interventions to increasingly routine, standard procedures. As less invasive procedures are used safely, effectively, and with greater frequency among older Americans to enhance well-being and longevity [4–6], both consumer demand for and the ethical pressure to offer them have increased. Second, norms of “old age” have shifted in US society and in clinical medicine [7,8]. Physicians described changing definitions of “how old is ‘old.’” In addition, physicians viewed old age not as a time of inevitable decline and death but as one of *preventable* morbidity and mortality, when clinicians must “look forward for” and “treat for risk.” Third, the framing of medical choices for elderly Americans and their physicians is shaped by the Medicare system, in which reimbursement is tied to procedures performed.

These three developments in cardiac medicine—reflective of wider changes throughout clinical medicine—produced an experience among the physicians we interviewed of an almost inexorable momentum towards treatment. One characterized the successive and escalating cardiac interventions often undertaken for very elderly people as a “speeding train.” Another called it an “extravaganza of cardiology” and a “technology parade,” in which a stream of cardiac specialists each performs a procedure that is medically indicated but also facilitated, in part, by those that preceded it.

**Summary Points**

- The “treatment imperative” refers to the almost inexorable momentum towards intervention that is experienced by physicians, patients, and family members alike.
- Even in the presence of reservations about the appropriateness of continued treatment, decisions regarding cardiac treatment in late life are often scripted to favor intervention.
- This technological ethic is driven by rising expectations for vitality and longevity, the growing availability of options for intervention, changing norms of old age, and the stark urgency presented by cardiac risks that are reducible through increasingly reliable procedures.
- The treatment imperative has become a standard of ethical clinical practice, and together with social changes and Medicare policies, contributes to an “extravaganza of cardiology” at ever older ages.
- However, we argue that clinicians can bring questions of ethics, quality of life, and the limits of cardiac intervention into better expression in their interactions with their patients, and we offer suggestions for how to do so.

Yet even while clinicians experienced this compulsion to treat, they also affirmed the need to consider the ethical appropriateness of a cardiac “full-court press” [9]. An interventional cardiologist cautioned, “I can get the patient through a lot, but you've got to think, what's your end-point?” A cardiac surgeon talked about the need to question whether meaningful life prolongation can be achieved with those patients hard-pressed to withstand the “march” through cardiology, catheterization lab, operating room, intensive care unit, and recovery facility.

Yet clinicians confided that they found it difficult to know how and when to pose such questions to their patients or to voice the doubts that underlie them. They attributed this difficulty, first, to their inability to prognosticate reliably which patients will experience positive, negative, or unchanged outcomes, particularly among the elderly [10]. As a result, practitioners said they based decisions not on whether patients would be well-served in the long term by a specific treatment, but largely on whether they were simply eligible for a procedure.

Second, clinicians perceived that their difficulty with prognostication in turn imposed an ethical injunction against raising questions about the appropriateness of pursuing therapies. Physicians believed that expressing any such doubts in the absence of reliable clinical predictions would be morally equivalent to substituting their own treatment preferences and values regarding quality of life for those of their patients [11,12]. As a cardiac surgeon put it, “You would *never* want to see a physician if you thought that for one second he was weighing whether or not your social worth and potential longevity are such that we should put this amount of resource into making you well.”

Third, our physician respondents reported avoiding discussions about meaningful life extension because of a phenomenon we called “technological incrementalism”: once treatment of any kind is initiated, each successive procedure is relatively easy to justify because it is seen to be only incrementally more or less risky than the previous one [1]. Such serial, piece-meal intervention is “hard to grind to a halt,” as one doctor put it, because there are no clear points in the treatment trajectory when it seems clinically or morally acceptable to refuse to move to that next step. The need to address the clinical exigencies of the moment mostly trumped reflection about the long-term aims of cumulative therapies and the consideration of comfort or palliative care as either an alternative or supplement to life-prolonging interventions.

Norms of “old age” have shifted in US society and in clinical medicine.

Clinicians instead reported feeling duty-bound to “err on the side of doing interventions,” “cover the bases,” and “give the patient the benefit of the doubt.” Clinicians viewed this as the only appropriate way to navigate through the complex thicket of prognostic ambiguity, personal judgments about quality of life, and the actual and perceived promise of medicine. As a result, the treatment imperative came to embody for physicians both a clinical standard of care as well as the definition of ethical responsibility.

## Unstated Options: The Consequences for Patients

Patients and families experienced the treatment imperative as well. Their desire to maximize quality of life and longevity, together with their sense of familial obligation, made it extremely difficult to forego medical options. But, in addition, patients rarely recalled any communication regarding potentially negative treatment outcomes, other than during informed consent procedures which many viewed as mandatory, cursory, and overly general. Most indicated that they were not offered the option of *no* treatment. One woman related that her physician told her baldly, “‘It's not a choice of whether you want to go through [treatment] or not if you want to live.’ When you faced with that kind of a ‘choice,’” this patient said, “your decision is made for you.”

We heard from patients that when their physicians did not explicitly present the option of *no* treatment, patients assumed that their doctors believed a positive outcome to be so likely that nonintervention was out of the question. The lack of any kind of countervailing or meaningful discussion regarding possible risks, negative outcomes, and death from the patients' perspectives led to their unqualified confidence in life-prolonging interventions [13]. Older individuals' expectations about post-procedure life therefore were shaped as much by what was left unsaid as by what was said.

Consequently, patients were often shocked and demoralized when treatment failed to help. While physicians understand unchanged or negative outcomes as being well within the range of the possible, such results led patients to question the value of their existence, and eroded the trust they placed in medicine and their doctors. “I'm utterly useless, a vegetable … I don't know what to do with myself,” sobbed one such patient. Another asked, contemplating her bleak future, “Do I have to continue this way? I would rather [my life] ended, but I'm not in a mood to commit suicide.”

## Reflections and Recommendations

Medical intervention in late life can be characterized by a tension between the desire for prolonged life and a wish for a dignified end. Conventional framings of this debate have usually resolved this tension, at least in the hypothetical, by invoking patient “autonomy” and shared decision-making [14–16]. By facilitating these values, it is suggested, patients will be able to decide for themselves whether to prolong life or allow death, and physicians will be able to adjust their recommendations to reflect patients' desires. But the ideal of patient choice and the discernment of patients' authentic values are extremely elusive [17,18].

The developments we describe have permeated the social fabric of our society and the organization and culture of medicine. And they have left us in a quandary. Physicians, patients, families, and the public may feel uneasy about a full-court press approach to clinical intervention at ever older ages. Yet it is difficult to imagine an alternative response to the treatment imperative. It feels naïve or overly reactionary to say “no” to the medical options that already exist, or to slow down or stop the technology parade, even if at times it feels more like a runaway train. Nor is it possible to imagine a future without expanding clinical choices. Our growing ability to extend life has become part of how we envision life itself and the malleability of our bodies and futures.

Our ethnographic evidence indicates that questions about death, quality of life, and the limits of intervention are often not expressed in patient–physician conversations about cardiac procedures. Given the structural and ethical pressures to offer and accept treatments, what can physicians do to better guide their older patients in making therapeutic decisions? Based on our findings, we offer two modest recommendations.

First, clinicians can make the option of accepting no intervention aimed solely at extending life an explicit one. Such an articulation might be the very entrée some patients, families, and physicians need in our procedure-driven system to bring their concerns regarding the appropriateness of life prolongation above ground. In so doing, physicians may feel more comfortable discussing symptom control and comfort measures, and patients and families may better grasp whatever added risks their health status and advanced age might pose—even if, in the end, they still find it impossible to forego treatment.

Second, clinicians can communicate more explicitly the full range of possible outcomes, and further, can attempt to describe the probable outcomes for that individual patient. Nicholas Christakis has written eloquently about the myriad reasons why physicians are “reluctant prophets,” and he offers numerous ideas for how doctors might more accurately formulate and communicate prognoses [10]. Our study suggests that patients tend to assume the positive when physicians do not state (in a manner that patients are likely to hear) the possibilities for negative results. Older individuals subsequently are demoralized by the unanticipated outcomes of no change or worsening health, which undermines their trust in medicine and their doctors, upon whom they must continue to depend.

We are well aware of the obstacles that stand in the way of making these kinds of improvements [19]. For example, it may be that physicians are sharing information that patients retrospectively believe would have been helpful, but patients do not apprehend it at the time. Sensitive conversations about values and visions for life near its end, which are already difficult to initiate and navigate, may be impossible to carry out in a meaningful way when time is short, as it is in most clinical settings today. Some, perhaps many, patients may abjure the responsibility of making medical decisions and so do not wish to participate in communication regarding them. Or it may be that no matter how much advance information patients are given, shock and disappointment are unavoidable when treatment fails to help. Finally, we must acknowledge that a health care and reimbursement system predicated on intervention can thwart the effects of any communicative efforts by individual physicians. Given human hope and the desire to live, it is difficult to expect patients and clinicians to sometimes say “no” when they are immersed in an organizational context and culture of hope that mostly says “yes.”

Clinicians can communicate more explicitly the full range of possible outcomes.

Despite such obstacles, we do not believe that any of the clinicians we spoke with, nor the larger medical community, would concede that the treatment imperative is inevitable, or that the mere existence of an intervention determines our use of it. However individual patients and doctors respond to the question of whether or not to intervene, it must remain just that: a question to be posed and a decision to be made, rather than a routine, normalized course of action, against which all other paths become increasingly inconceivable and unavailable. In an era in which we desire and believe in patient choice as an ideal—even if we struggle with its actual consequences for clinical practice and human experience—it would be an ultimate irony if alternative choices to the treatment imperative were increasingly narrowed.
